# Examination of Hematological Parameters in Patients with First-Episode Psychosis within the Bipolar Disorder or Schizophrenia Spectrum

**DOI:** 10.1192/j.eurpsy.2025.831

**Published:** 2025-08-26

**Authors:** E. Uzun Uysal, Ö. A. Uysal, B. İnce

**Affiliations:** 1 Arnavutköy State Hospital; 2 Bakırköy Training and Research Hospital for Psychiatry, Neurology and Neurosurgery, İstanbul, Türkiye

## Abstract

**Introduction:**

First-episode psychosis (FEP) provides a crucial opportunity to investigate these biological markers before the influence of long-term treatment or disease progression. Both bipolar disorders with psychotic features and schizophrenia spectrum disorders are linked to systemic inflammation, and examining blood cell counts and inflammatory ratios may provide insights into the biological foundations of these conditions.

**Objectives:**

This study aims to compare key hematological parameters and inflammation markers (neutrophil/lymphocyte ratio (NLR), platelet/lymphocyte ratio (PLR), monocyte/lymphocyte ratio (MLR)) between untreated first-episode psychosis patients diagnosed with psychotic mania, schizophrenia spectrum disorders, and healthy controls, examining potential differences between these groups.

**Methods:**

55 patients (F:28, M:27) diagnosed with schizophrenia spectrum disorder, 68 patients (F:38, M:30) diagnosed with bipolar disorder, all without a history of treatment, who were admitted to psychiatric clinics due to a first-episode psychosis, and 61 healthy volunteer individuals (F:24, M:37) matched for age and gender were included in the study. Hemogram data were obtained from medical records, and the hemograms taken within the first 48 hours of the patients’ admissions were used in the study.

**Results:**

The white blood cell, neutrophil, and monocyte counts were significantly higher in both patient groups than healthy individuals. The eosinophil count varied between the groups, with patients diagnosed with schizophrenia spectrum disorder having significantly lower counts compared to healthy individuals (p=0.003). When the analysis was conducted by gender, white blood cell, neutrophil, and monocyte counts were found to differ in women from both patient groups compared to healthy individuals, while in men, only the eosinophil count was lower in patients diagnosed with schizophrenia spectrum disorder (p=0.023). There were no significant differences in the NLR and PLR between the groups. The MLR value showed no difference between male patients and healthy individuals, but it varied between the groups in women, with patients diagnosed with psychotic mania having higher MLR compared to the other groups (p=0.01).

**Conclusions:**

Changes observed in specific hematological parameters in both bipolar disorder and schizophrenia spectrum patients may contribute to understanding the pathophysiology of these disorders. However, given the heterogeneity in the presentation and etiology of these conditions, larger-scale and prospective studies are needed to determine the roles of these parameters in their pathophysiology. It may also be necessary to consider gender-based differences when assessing the potential roles of these hemogram parameters in the pathophysiology of the diseases.

**Disclosure of Interest:**

None Declared

